# Eating Habits, Physical Activity and Nutritional Status Among Kenyatta University Students

**DOI:** 10.1155/jnme/9951472

**Published:** 2025-11-29

**Authors:** Joseph Mburu Ndung'u, Joseph Kobia, Judith Waudo

**Affiliations:** Department of Food, Nutrition & Dietetics, Kenyatta University, Nairobi 00100, Kenya

**Keywords:** eating habits, obesity, overweight, physical activity, sedentary lifestyle, university students

## Abstract

**Introduction:**

Obesity has become a global health crisis, with prevalence nearly tripling since 1975. In 2016, approximately 39% of adults worldwide were overweight or obese. Lifestyle factors, such as dietary habits and physical activity levels, significantly contribute to this alarming trend. This study aimed to assess the eating habits, physical activity levels and nutritional status of university students.

**Material and Methods:**

This was a cross-sectional analytical study. The study was conducted among 249 students from 17 schools within the university, selected using a multistage stratified sampling technique. Data analysis was performed using SPSS version 24.0.

**Results:**

The study participants were aged between 18 and 31 years. The findings revealed that 79.9% of students exhibited a mixture of both healthy and unhealthy eating habits, whereas 8% and 12% demonstrated unhealthy and healthy eating habits, respectively. A higher percentage of females (70%) had more nutritious eating habits compared to males. Overall, 75.9% of students were classified as physically inactive. A higher proportion of females (61%) were physically inactive compared to males (39%). In comparison, 49.8% reported sitting for eight or more hours per day, indicating a sedentary lifestyle that further contributes to health risks. Based on BMI, 8.4% of students were underweight, 67.5% were within the normal range, 16.5% were overweight and 7.6% were obese. However, based on waist circumference, 21.7% exhibited abdominal obesity. Furthermore, females had significantly higher BMI and waist circumference values. Statistical analysis revealed significant associations between BMI and age, year of study and meal source. Waist circumference was significantly associated with sex, year of study and residence status. Additionally, a significant relationship was observed between residential status and eating habits, and the daily consumption of fast food was significantly associated with BMI.

**Conclusion:**

It is imperative to consider gender-specific interventions to address physical inactivity, obesity and abdominal obesity among university students. Future research should utilise objective methods to assess physical activity levels for a more comprehensive understanding of student health.

## 1. Introduction

In 2022, there were over 2.5 billion adults who were overweight worldwide and 890 million with obesity representing 43% and 16% of all adults, respectively [1]. Obesity is already responsible for more deaths worldwide than smoking [2]. Students in higher education, particularly young adults on the cusp of independent life, are at a crucial stage to establish habits that will remain with them throughout their lives [3]. Status of female sex, age, chronic disease, high frequency of intake of soft drinks, physical inactivity and usage of motor transport have been linked to a high risk of obesity in Ethiopia [4], whereas in Kenya, urban residency, increased socioeconomic status, cohabiting and education status have also been revealed with similar linkages [5]. Life in universities is thus a critical setting in determining food and lifestyle choices. Research has identified everyday unhealthy diet and eating patterns among students, including frequent meal skipping, inadequate intake of fruits and vegetables and excessive fast-food consumption [6], which results in an estimated 36% or more overweight and obesity incidence [7]. Physical inactivity also comes under the lens. Yun et al. [8] documented that 25.4% of the students exercised habitually, and Ekelund et al. [9] linked prolonged sedentary time (eight or more hours daily) with a 59% higher risk of death at age 59. Such unhealthy behaviour, if formed in childhood, is sure to be carried into adulthood. In Kenya, findings show alarming tendencies: Nyanchoka et al. [10] had previously quoted the prevalence of overweight and obesity as 13.9% and 4.3%, respectively, but Mwangi et al. [11] had quoted higher percentages of 31.2% for overweight and 6.2% for obesity among undergraduate students, with higher prevalence among females.

Although existing studies have focused on lifestyle and diet among university students, gaps still need to be addressed regarding the dietary intake, physical activity and the respective determinants among Kenyan university students. University life is a time of transition in food and level of activity that will most likely influence health. There is not much, however, on how demographic and socioeconomic determinants shape such behaviours in Kenyan students. The current study aimed to evaluate lifestyle habits and nutritional status among Kenyatta University undergraduate students. It considered their demographic and socioeconomic factors, eating habits, physical activity and nutritional status and looked at the interrelation among them.

## 2. Materials and Methods

### 2.1. Study Design and Setting

This study employed a cross-sectional analytical design. The target population comprised undergraduate students enrolled at Kenyatta University Main Campus, Nairobi County, Kenya.

### 2.2. Study Variables

#### 2.2.1. Independent Variables

• Demographic Factors: Age, gender, year of study, residence and programme of study
• Lifestyle Factors: This broad category encompasses a range of behaviours:
  ◦ Eating Habits: Consumption of healthy and unhealthy foods, monitoring and planning for consumption of healthy foods

Eating habits were measured and classified using a tertile classification system. This system is widely used in dietary pattern research [12]. For this study, the nutritional habit scores were aggregated and classified as follows:

• Lower tertile, including those whose scores were in the bottom 33%: Unhealthy eating habits
• Middle tertile included those in the middle 33% (87–135): Moderate eating habits
• Upper tertile, including those in the approximately top 33% (136–185): Healthy eating habits
  ◦ Physical Activity Patterns: Levels of physical activity

#### 2.2.2. Dependent Variable

Nutritional Status: This was assessed using two key indicators. Anthropometric measurements were taken by trained personnel according to standardised protocols to ensure accuracy and reliability. Weight was recorded on calibrated digital scales to the nearest 0.1 kg, and height was recorded using stadiometers to the nearest 0.1 cm. Body mass index (BMI) was calculated as weight in kilogrammes divided by height in metres squared (kg/m^2^). Waist circumference (WC) was measured halfway between the lowest rib and the iliac crest with a nonstretching measuring tape to the nearest 0.1 cm.

### 2.3. Sampling and Sample Size

A multistage stratified sampling technique, followed by simple random sampling, was used to select a sample of 249 students from the 45,348 active students registered in the 2020/2021 academic year. This accounted for a 95.77% response rate as the planned sample size was 260 students. All 249 cases were included in the data analysis.

### 2.4. Data Quality Control

The research instrument was subjected to nutrition experts, including thesis supervisors, for review to determine its validity. To ensure the accuracy and reliability of the data, several quality control measures were implemented. Firstly, a pretest was conducted on 10% of the target population (*n* = 26) to assess the reliability of the research instruments. Test–retest reliability was evaluated, with a correlation coefficient above 0.7 considered acceptable [13]. This step aimed to confirm the consistency of the instruments in producing similar results when administered to the same individuals at different times.

### 2.5. Data Analysis

Data were entered and analysed using SPSS version 24.

• Eating Habits:
  ◦ Eating habit scores were aggregated and categorised as follows:
• 37–86: Unhealthy eating habits
• 87–135: Moderate eating habits (a combination of healthy and unhealthy eating habits)
• 136–185: Healthy (adapted from [12])
• Physical Activity:
  ◦ Sufficient: Meeting the recommended minimum of 150 min per week of moderate-intensity activity or 75 min of vigorous-intensity activity per week.
  ◦ Insufficient: Below the recommended minimum of 150 min per week of moderate-intensity activity or 75 min of vigorous-intensity activity per week [14].
• Sedentary Lifestyle:
  ◦ Defined as sitting down for ≥ 8 h per day [15].
• Nutritional Status:
  ◦ Nutritional status was assessed using BMI with the following classifications:
    - < 18.5 = underweight
    - 18.5–24.9 = normal
    - 25–29.9 = overweight
    - ≥ 30 = obese [1]
  ◦ Central Obesity:
    - Central obesity was defined as a WC ≥ 80 cm for women and ≥ 94 cm for men [1].

### 2.6. Ethical Consideration

Ethical clearance for this study was granted by the Kenyatta University Ethical Review Committee (Reference Number: PKU/2108/1108). Before commencing data collection, all participants were provided with written informed consent documents outlining the research's purpose and objectives. To safeguard participant confidentiality, personal names were replaced with unique identifying codes.

## 3. Results

### 3.1. Demographic and Socioeconomic Characteristics of Undergraduate Students at Kenyatta University

Table 1 presents the demographic and socioeconomic characteristics of the 249 undergraduate students at Kenyatta University. The majority of participants were female (61%), with most falling within the 18–24 age group (85.1%). The student body was diverse, with representation from various academic years, including Year 1 (26.1%), Year 2 (7.2%), Year 3 (17.7%) and Year 4 (49%). The School of Education had the highest representation (34.1%), followed by the School of Humanities and Social Sciences (16.9%). Regarding residence, 51% of students resided off-campus in their own houses, whereas 25.3% lived on campus. Self-preparation was the primary source of meals for 69.5% of students, followed by restaurants (19.3%) and university cafeterias (11.2%).

### 3.2. Eating Habits of Undergraduate University Students

#### 3.2.1. Unhealthy Eating Habits of Undergraduate University Students

Table 2 provides an in-depth examination of the frequency of various dietary behaviours among 249 undergraduate students. Notably, a significant proportion of respondents reported frequent consumption of fast food (38.6% 1–2 times/week), aligning with a concerning trend of high consumption for fatty (40.6%) and fried foods (28.1%–40.6%). The high consumption of fast, fatty and fried foods may have implications for student health, potentially increasing the risk of chronic diseases. Although 36.5% of students never consumed desserts, a substantial portion (32.5%) reported consuming them 1–2 times per week. Restaurant meals were also a common dietary component, with 30.5% of respondents consuming them at this frequency.

Beverage habits revealed a strong preference for tea (47.4%) and coffee (32.5%) with meals. Snacking between meals was reported by 20.5% of students 1–2 times per week. Daily consumption was observed for salty foods (33.3%) and spicy foods (16.1%) in a considerable number of respondents. Conversely, less frequent habits included consuming undercooked foods (55%), canned foods (78.7%) and drinking fizzy drinks with meals (30.5%).

#### 3.2.2. Healthy Eating Habits of Undergraduate University Students

Table 3 presents the frequency of reported healthy eating habits among the 249 undergraduate students. Encouragingly, a substantial majority (58.2%) consumed breakfast daily. Furthermore, a high proportion of students reported increased consumption of fruits (51.8%) and vegetables (32.9%). Daily dairy intake was reported by 28.9% of participants. The high frequency of fruit, vegetable and water consumption suggests a generally positive approach to healthy eating among the student population.

Although 33.3% of students consumed whole-grain foods daily, the data revealed a lower frequency of low-fat salad consumption, with 6% of students consuming it. A notable finding was that 45% of students reported eating breakfast purposefully, suggesting an awareness of its nutritional importance. Consistent meal patterns were observed, with 35.7% of students reporting consuming three meals daily. Finally, 28.1% of students reported adhering to a nutritious diet daily, whereas 3.6% never did, indicating a range of dietary practices within the student population. These findings suggest a general positive trend towards healthy eating practices among the undergraduate student population.

#### 3.2.3. Monitoring Healthy Eating Habits of Undergraduate University Students

Table 4 reveals a notable gap in dietary tracking practices among the undergraduate student population. A substantial proportion (50%) did not monitor their food intake over a week, suggesting a potential lack of awareness regarding the significance of dietary tracking for informed nutritional decision-making. Although 21.7% of respondents tracked their breakfast intake daily, 59% never did, highlighting a potential area for improvement. Furthermore, the frequency of tracking key dietary components was generally low. Only 10% monitored nutrient composition daily, and tracking of soda intake (16.9%) and fruit/vegetable consumption (9.6%) was similarly infrequent. Notably, a large majority (69.9%) never tracked their whole-grain intake. These findings suggest a need for interventions to enhance dietary tracking practices among this population, potentially through educational initiatives and the provision of accessible tracking tools. The low frequency of tracking suggests that many students may not be actively monitoring their dietary choices. This suggests a potential gap in awareness and utilisation of dietary tracking tools and techniques to support healthy eating behaviours. Possible barriers to tracking could include a lack of time, motivation or knowledge about how to track dietary intake effectively.

#### 3.2.4. Meal Planning Practices for Eating Healthy Foods of Undergraduate University Students

Table 5 provides insights into how often respondents planned for healthy eating behaviours. Notably, a substantial proportion of students reported limited proactive planning. A significant percentage (45%) never planned for breakfast intake, and a similar trend was observed for planning to consume five servings of fruits/vegetables (54.6%) and three servings of dairy foods (58.2%). Although a smaller proportion of students planned for these aspects more frequently, a notable finding was that 23.7% planned to consume nutritious meals daily, suggesting some level of proactive dietary consideration. These findings underscore the need for interventions that emphasise the importance of meal planning as a crucial strategy for promoting and sustaining healthy eating habits among university students. This finding suggests that many students may not actively plan their meals, such as by meal prepping and grocery shopping, to ensure they are making healthy choices.

#### 3.2.5. Eating Habit Scores of Undergraduate University Students

An aggregate score was calculated to assess overall eating habits, encompassing unhealthy, healthy and related behaviours. Scores were categorised as unhealthy (37–86), moderate eating (those who inhibited a combination of both healthy and unhealthy eating habits) (87–135) and healthy (136–185), as shown in Table 6. Analysis revealed a mean eating habit score of 110 ± 20.5, indicating a distribution with a substantial proportion of students falling within the ‘neutral' category. Overall, 79.9% of respondents exhibited neutral eating habits, followed by 12% with healthy habits and 8.0% with unhealthy habits.

### 3.3. Physical Activity Level of Undergraduate University Students

Table 7 reveals a concerning level of physical inactivity among the undergraduate student cohort. A substantial proportion (75.9%) did not meet the recommended 150 min of moderate-intensity physical activity per week. Notably, a higher proportion of 18–24-year-olds (84%) were classified as inactive compared to their older counterparts. This suggests that younger students may be less engaged in physical activity. Females exhibited a higher prevalence of inactivity (64%) compared to males (36%). A significant proportion (49.8%) reported engaging in sedentary behaviour for 8 h or more per day.

### 3.4. Nutritional Status of University Students

#### 3.4.1. Nutritional Status of University Students Based on BMI Classification

The average weight and height of the respondents were determined to be 62.7 ± 11.3 kg and 1.6 ± 0.1 m, respectively. Analysis of BMI revealed that 8.4% of the respondents were underweight, 67.5% were within the normal range, 16.5% were overweight and 7.6% were obese. The combined prevalence of overweight and obesity (BMI ≥ 25) was 24.1%. Gender disparities were observed in BMI distribution. A higher percentage of males (57.1%) were classified as underweight compared to females (42.9%). Conversely, a higher percentage of females (63.3%) were classified as obese compared to males (36.8%).

#### 3.4.2. Nutritional Status of University Students Based on WC Classification

Analysis of WC revealed that 21.7% of the respondents exhibited central or abdominal obesity, whereas 78.3% did not. The average WC was 77.8 ± 9.7 cm, suggesting that the majority of students were not at a significantly high risk of abdominal obesity. Gender disparities were evident, with a higher prevalence of abdominal obesity observed in females (87%) compared to males (13%). Age-related differences were also observed. In the 18–24 age group, 20.3% exhibited abdominal obesity, whereas this figure increased to 29.7% in the 25–31 age group, suggesting a potential increase in abdominal obesity with advancing age. Interestingly, the prevalence of central obesity (based on WC) was lower than the combined prevalence of overweight and obesity (based on BMI), which was 24.1%. This discrepancy may be attributed to the limitations of BMI, which can overestimate obesity risk in individuals with high muscle mass. These findings underscore the importance of considering WC in addition to BMI for a comprehensive assessment of obesity risk, particularly in populations with varying levels of physical activity.

### 3.5. Associations Between Demographic Factors, Socioeconomic Status, Eating Habits, Physical Activity and the Nutritional Status of Undergraduate University Students

#### 3.5.1. Associations Between Demographic Factors, Eating Habits and BMI

Residence status had a significant association with eating habits (*p*=0.020). Analysis of variance confirmed this finding, demonstrating significant differences in mean eating habit scores across different residence categories (*p*=0.005; df = 3; *F* = 4.318). Notably, students residing on campus exhibited the highest mean eating habit scores. Occasional fast-food consumption was strongly associated with an increased likelihood of obesity compared to individuals who never consumed fast food (*p* < 0.001; OR = 2.725E + 8). This association was even stronger for individuals who consumed fast food daily (*p* < 0.001; OR = 3.893E + 8). This is evidenced by the extremely high odds ratio (OR = 3.893E + 8) and the highly statistically significant *p*-value (< 0.001). Interestingly, rare fast-food consumption was associated with a significantly increased likelihood of maintaining a normal BMI (*p* < 0.001; OR = 3.406) compared to those who never consumed fast food.

Age was found to be a significant predictor of BMI (*p*=0.001). In particular, students aged 18–24 years were nearly four times more likely to have a normal BMI compared to those aged 25–31 years (OR = 3.929; 95% confidence interval [CI]: 1.233–12.513). Furthermore, a significant positive correlation was observed between age and BMI (*p*-value = 0.024; *r* = 0.143), indicating that BMI tended to increase with age among this student population. The year of study also demonstrated a significant association with BMI (*p*=0.0049), suggesting that nutritional status may vary across different academic years. Students in their first year of study were found to be 11 times more likely to have a normal BMI compared to those in their fourth year (*p*-value = 0.020; OR = 11.397; CI = 1.465–88.653). This suggests a potential decline in nutritional status as students progress through their university education.

Adjusted logistic regression analysis revealed a significant association between sex, year of study and BMI. Males were found to be significantly five times likely to be underweight compared to females (*p*-value = 0.030; AOR = 4.956; 95% CI: 1.168–21.028). This finding suggests that sex may play a significant role in influencing BMI and the risk of underweight among university students. Notably, first-year students exhibited a markedly higher likelihood of maintaining a normal nutritional status compared to their fourth-year counterparts (AOR = 13.411; 95% CI: 2.691–120.727; *p*=0.021). This observation was further supported by a significant elevation in the likelihood of normal BMI among third-year students relative to fourth-year students (AOR = 11.803; 95% CI: 1.231–113.126; *p*=0.032). These findings suggest a potential decline in nutritional status as students progress through their academic journey, particularly during the transition to the final year of study.

The source of meals was significantly associated with BMI (*p*=0.021), highlighting the potential influence of dietary patterns on nutritional status. In particular, students who frequently ate at the university cafeteria were 0.221 times less likely to have a normal BMI compared to those who frequently ate at restaurants (*p*-value = 0.036; OR = 0.221; CI = 0.039–1.240).

#### 3.5.2. Associations Between Demographic Factors, Physical Activity and BMI

Our findings revealed that the programme of study (*p*=0.032) and residential status (*p*=0.012) had a significant relationship with physical activity. Students pursuing creative and performing arts demonstrated a considerably lower likelihood of physical inactivity compared to those in education programmes (*p*=0.025; OR = 0.325; 95% CI: 0.122–0.867). This finding aligns with the inherent physical demands of these programmes, encompassing disciplines such as dance, music and theatre. Similarly, students in pure and applied sciences programmes exhibited a significantly reduced risk of physical inactivity compared to their counterparts in education programmes (*p*=0.010; OR = 0.167; 95% CI: 0.042–0.657).

Furthermore, residing at home with family was associated with a significantly lower probability of physical inactivity compared to living independently off-campus (*p*=0.044; OR = 0.410; 95% CI: 0.172–0.975). In contrast, factors such as sex (*p*=0.096), age (*p*=0.534), year of study (*p*=0.696) and source of meals (*p*=0.082) did not exhibit significant relationships with physical activity levels within this student population.

#### 3.5.3. Demographic Factors, Eating Habits, Physical Activity and WC

The study found a statistically significant association between eating habits and WC (*p*=0.044). Individuals with healthy eating habits were nearly three times more likely to have a normal WC compared to those with neutral eating habits (*p*-value = 0.015; OR = 2.735; CI = 1.217–6.148). This suggests that adopting healthy eating patterns can significantly reduce the risk of abdominal obesity among university students. Physical activity (*p*=0.997) was not found to have a significant association with abdominal obesity among the student population.

Sex (*p*=0.001), year of study (*p*=0.027) and residence status (*p*=0.040) were significantly associated with central obesity. Males were nearly six times likely to have a normal WC compared to females (*p*-value = 0.001; OR = 5.755; CI = 2.478–13.364). This suggests that males may have a lower risk of abdominal obesity compared to females. Third-year students were significantly more likely to have a normal WC compared to fourth-year students (*p*-value = 0.022; OR = 3.265; CI  = −1.190–8.956).

Students living off-campus in hostels were found to be three times more likely to have a normal WC compared to those residing off-campus in their own houses (*p*-value = 0.048; OR = 3.093; CI = 0.881–10.854). This suggests that living in a hostel environment may be associated with a lower risk of abdominal obesity. Hostel environments may provide more opportunities for physical activity, healthier meal options or social support for healthy lifestyles compared to living independently in rented houses.

Adjusted logistical regression showed that sex and year of study had significant associations with WC. Males were eight times more likely to have a normal nutrition status (WC) as compared to females (*p*-value < 0.001; AOR = 8.018; CI  = −3.190–20.151). First-year students were significantly more likely to have a normal WC compared to fourth-year students (*p*-value = 0.036; OR = 2.302; CI = 1.057–5.013). This suggests a potential increase in the risk of abdominal obesity as students progress through their university education. These findings highlight the importance of considering factors such as sex and year of study when assessing and addressing the risk of abdominal obesity among university students.

## 4. Discussion

### 4.1. Associations Between Demographic Factors, Eating Habits and Nutritional Status of Undergraduate University Students

This study found an association between on-campus residence and healthier eating habits. This finding aligns with previous research [16] that demonstrated a correlation between increased distance from the university and unhealthy eating behaviours. This trend is attributed to on-campus students having greater access to university-subsidised cafeterias that offer well-nourished food at minimal cost, along with a structured eating environment that promotes regular eating patterns. The on-campus environment removes the transportation constraints and time pressures that force off-campus students to resort to convenient but less nutritious fast-food alternatives. Prior studies [17] have highlighted the influence of education level and age on food choices. For instance, research among Iranian students [18] revealed a higher likelihood of fast-food consumption (pizza and fried chicken) among females. Furthermore, another study [19] observed a link between unhealthy dietary intake (fried chicken and pastries) and males, whereas energy drink consumption was more prevalent among females.

Our study found that advanced age, being in the fourth year of study and frequenting restaurants were associated with a higher BMI. Adjusted logistic regression analyses revealed that being female and in the fourth year of study were significant predictors of higher BMI, whereas being male and in the first year of study increased the likelihood of being underweight. These findings resonate with previous research. These findings imply that preventive interventions must be directed at students early during university life, preferably in orientation and first-year programmes, before weight problems become entrenched. Universities must implement year-specific wellness programmes and enhance campus food environments. Meanwhile, public health officials can investigate regulating the density of fast-food outlets around campuses and inserting nutrition education into core curricula. A study in Peru [20] found that overweight and obesity were significantly associated with advanced age (> 27 years) and pursuing engineering studies but not with gender. López-Moreno et al. [21] reported a significant association between gender and body weight. Furthermore, studies among Cameroon medical students [22] and Serbian nationals [23] found significant associations between BMI and factors such as gender, age and education level. Consistent with our findings, [24] observed lower BMI values in males compared to females.

Nevertheless, other studies reported an insignificant relationship between age, year of study, source of meals and BMI. Studies conducted in Iran [25] and Canada [19] found that being male increased the risk of higher BMI among undergraduate students. This finding is supported by [23], who observed a significant association between overweight and obesity and the male gender among Bangladeshi university students. Furthermore, [26] found that residential status was correlated with BMI among Rwandese university students.

This study found that being female, in the fourth year of study and residing off-campus in one's own house were associated with higher WC. Adjusted logistical regression confirmed that female sex and the fourth year of study were significant predictors of increased WC. The fact that off-campus independent living is associated with greater WC probably mirrors budget constraints that put rent ahead of food quality, with the effects of having to survive on cheaper, energy-dense processed food. Moreover, a lack of environmental structures for meals and rudimentary cooking skills can lead to irregular eating patterns and increased consumption of convenience foods high in refined carbohydrates and fat. A Kenyan study by [27] specifically concluded that females had significantly higher WC compared to males.

On the contrary, [28] in Iran and [19] in Canada found that male sex was associated with increased WC risk among undergraduate students. A study by [25] found no significant associations between sex, year of study and living with family and WC. Similarly, a study by [29] among University of Botswana students found no significant associations between gender, programme of study and obesity.

The present study observed a significant association between BMI and eating habits, specifically the consumption of fast foods. This finding aligns with a growing body of global research. Reference [30] identified frequent consumption of meals, junk food, fast food and soft drinks as significant risk factors for obesity and overweight among Bangladeshi university students. This aligns with the findings of [29], who demonstrated a substantial link between unhealthy dietary practices and overweight/obesity among Botswana University students. Although [18] observed an association between fast-food intake (pizza, sandwiches and fried chicken) and abdominal obesity, this association was not evident with general obesity as measured by BMI. Extending these findings, [26] in Rwanda reported that the weekly frequency of fast food, vegetable, fruit, chicken and snack intake was significantly associated with the BMI of university students. The results provide evidence to inform national policy interventions in the university food environment, such as sugar-sweetened beverage tax, compulsory nutritional labelling in cafeterias and banning unhealthy food marketing to students. Universities should be designated as priority settings for obesity prevention, given the concentration of young adults and the potential for establishing lifelong dietary patterns. The present study found that individuals who followed healthy eating habits were substantially more likely to have a normal WC compared to those with average eating habits. This finding is supported by previous research conducted by [19] in Canada, which demonstrated a positive association between WC and the consumption of saturated fats, sodium and pizza.

### 4.2. Associations Between Demographic Factors, Physical Activity Level and the Nutritional Status of Undergraduate University Students

Our findings showed that programmes of studies (such as creative and performing arts) and residing off-campus in one's own house were associated with an increased likelihood of physical inactivity. Students in the arts might have irregular schedules with night-time rehearsals and studio work that interfere with regular gym hours and their artistic work, although strenuous, might not be characterised by prolonged moderate-to-vigorous physical activity. Off-campus residents likely experience increased sedentary commuting time, lack affordable access to university recreational facilities and face time constraints from domestic responsibilities that compete with exercise opportunities. This finding aligns with previous research by [31], which reported that living in urban areas was associated with reduced physical activity in Bangladesh. Several studies have highlighted the significant influence of gender, sex and year of study on physical activity levels, but these factors were not significant in the present study. Studies by [32–34] found that being female and advancing age were associated with a decreased likelihood of engaging in physical activity.

This study found no significant association between physical activity levels and BMI or WC. This finding aligns with previous research. For instance, [25] in Saudi Arabia observed no significant relationship between physical activity engagement, duration, type and TV viewing with BMI or WC among university students. Similarly, an Asian study [35] found no association between physical inactivity, sedentary lifestyle and obesity. Universities need to create targeted interventions for groups at high risk by keeping recreational facilities open longer, providing subsidised membership for off-campus students and implementing discipline-specific fitness programmes that cater to students' unpredictable schedules. Satellite fitness centres in off-campus locations and peer-led activity groups may enhance access and participation for underserved student groups.

The lack of relationship between physical activity and nutrition status implies that obesity control cannot depend on the promotion of exercise alone but needs to be based on broad multicomponent programmes with equal focus on activity and diet. Public health planners will need to prioritise nutrition education and food environment change as the most critical interventions, accepting the seeming primacy of dietary influences on student weight status. Other studies have reported contrasting findings. In Syria, [36] observed a higher likelihood of high physical activity among males, whereas [30] in Bangladesh found that physical inactivity is significantly associated with obesity and overweight among university students, with 60% of those with low physical activity having a higher likelihood of these conditions.

## 5. Conclusion

This study illustrates concerning trends in undergraduate student nutritional status, including increasing BMI and WC across academic years, as well as high rates of poor dietary behaviours and low physical activity. Despite some food welfare among on-campus students, the high frequency of fast-food intake, meal skipping and socialising together among all students, along with notable gender differences in physical activity, mandates urgent intervention.

The findings provide a clear roadmap for evidence-based interventions. The universities would (1) redefine food environments on campus by mandating that 60% of food sold in cafeterias be nutritionally valuable and lowering the cost of healthy foods; (2) initiate mobile app–based meal-skipping interventions with personalised reminders and meal planning; (3) develop gender-sensitive physical activity programmes group activities for women and competitive sport for men; and (4) initiate year-specific programmes, that is, for first-year students during orientation before unhealthy behaviours become established. A comprehensive strategy must address individual behaviour, institutional policy and environmental factors simultaneously.

Following the findings from this study, interventions will have to be introduced in phases. Pilot interventions will be launched in targeted residence halls and departments. Success will also depend on introducing nutrition courses as general education requirements, starting peer health ambassador programmes, enforcing university policy on food vendor standards and keeping recreational facilities open later during peak stress times. Monitoring dietary intake, activity and anthropometric measures will hold everyone accountable and inform programme improvement.

Given the constraints of cross-sectional data and potential self-reporting bias, longitudinal studies must track students from matriculation through graduation to identify critical points of intervention and causal mechanisms. Mixed-methods studies must examine the impact of specific campus ecologies, social networks and academic stress on health behaviour. At the same time, randomised controlled trials assess the effectiveness and cost-effectiveness of interventions in diverse institutional settings.

Colleges and universities must move beyond awareness campaigns to implement complex, evidence-based interventions, supported by dedicated funding and institutional policy formation. By shaping healthful food environments, offering targeted education and physical activity programming and addressing gendered needs, higher education institutions can powerfully influence student health trajectories. The trends revealed in this study demand action today. Students' long-term health and well-being depend on concerted, urgent institutional action.

## Figures and Tables

**Table 1:** Demographic and socioeconomic characteristics of the respondents.

Variable	Male	Female	Total	%
Sex	97	152	249	100
Age (years)				
18–24	78	134	212	85.1
25–31	19	18	37	14.9
Year of study				
Year 1	23	42	65	26.1
Year 2	09	09	18	7.2
Year 3	10	34	44	17.7
Year 4	55	67	122	49.0
Programme of study				
Humanities and social sciences	11	31	42	16.9
Creative and performing arts	10	13	23	9.2
Pure and applied sciences	07	03	10	4.0
Hospitality, tourism and leisure	07	11	18	7.2
Education	28	57	85	34.1
Public health and applied sciences	11	12	23	9.2
Business	11	11	22	8.8
Economics	06	04	10	4.0
Agriculture and enterprise development	03	02	05	2.0
Environmental studies	00	06	06	2.4
Engineering and technology	03	02	05	2.0
Residence status				
On-campus	27	36	63	25.3
Off-campus (hostel)	16	17	33	13.3
Home with family	05	21	26	10.4
Off-campus (own house)	45	78	127	51.0
Source of meals				
Own preparation	59	114	173	69.5
University cafeteria	13	15	28	11.2
Restaurant	25	23	48	19.3

**Table 2:** Frequency of respondents' eating habits per week.

*n* = 249	Never	1–2 times	3–4 times	5–6 times	Daily
	%	%	%	%	%
Ate fast food	19.7	38.6	23.7	9.2	8.8
Ate fatty food	19.7	40.6	20.5	11.2	08
Ate fried foods	16.5	28.1	18.1	16.1	21.3
Ate desserts	36.5	32.5	13.7	8.4	8.8
Ate food from a restaurant	18.9	30.5	16.9	14.5	19.3
Drunk fizzy with meals	30.5	34.5	18.5	7.2	9.2
Drunk tea with meals	52.6	12	10.8	04	20.5
Drunk coffee with meals	67.5	12.9	10.4	2.8	6.4
Ate snacks between meals	30.5	20.5	22.9	10.8	15.3
Ate salty foods	22.1	23.7	14.1	6.8	33.3
Ate undercooked foods	55	20.5	11.6	06	6.8
Ate canned foods	78.7	16.5	02	02	0.8
Ate spicy foods	41	16.5	18.5	08	16.1

**Table 3:** Frequency of respondents' healthy eating habits per week.

*n* = 249	Never	1–2 times	3–4 times	5–6 times	Daily
	%	%	%	%	%
Ate breakfast	4.4	9.6	15.3	12.4	58.2
Ate 4–5 servings of vegetables	02	10	17.7	18.5	51.8
Ate more than 4–5 servings of fruits	04	10	35.7	17.3	32.9
Ate/drank more low-fat dairy	13.3	19.3	24.1	14.5	28.9
Ate whole-grain foods	14.5	16.1	17.7	18.5	33.3
Ate a low-fat salad	49.8	17.3	14.5	12.4	06
Ate breakfast purposefully	15.3	12.9	14.5	12.4	45
Ate three meals every day	8.4	16.9	19.3	19.7	35.7
Ate a nutritious diet	3.6	10.8	26.1	31.3	28.1

**Table 4:** Frequency of respondents' monitoring healthy eating habits per week.

Kept track of	Never	1–2 times	3–4 times	5–6 times	Daily
*n* = 249	%	%	%	%	%
No. of meals	71.9	6.0	4.4	7.2	10.4
Nutrient composition	73.1	6.8	5.2	4.8	10
Eating breakfast	59	6.0	6.4	6.8	21.7
Drinking sodas	57.4	9.6	6.8	9.2	16.9
Fruits/veggies intake	69.5	6.8	6.0	8.0	9.6
Dairy foods taken daily	72.7	8.8	4.8	5.2	8.4
Whole grain eaten	69.9	7.6	7.6	6.0	8.8
Fizzy drinks daily	73.9	5.2	5.2	5.2	8.8

**Table 5:** Frequency of respondents' planning for eating healthy foods.

Planned for	Never	1–2 times	3–4 times	5–6 times	Daily
*n* = 249	%	%	%	%	%
Breakfast intake	45	7.2	9.2	08	30.5
Five servings of fruits/veggies	54.6	9.6	7.6	8.8	19.3
Three servings of dairy foods	58.2	10.4	7.2	6.8	17.3
More whole grains	57	8.8	6.4	9.2	18.5
Fewer sodas	57.4	9.6	6.4	8.8	17.7
Three meals per day	48.2	6.8	11.6	7.6	25.7
Take nutritious meals	43.4	7.2	15.3	10.8	23.7

**Table 6:** Frequency of respondents' eating habits categories.

Eating habits					
Variable	Unhealthy	Neutral	Healthy	*n*	%
Age (years)					
18–24	19	167	26	212	85.1
25–31	01	32	04	37	14.9
Total	20	199	30	249	100
Sex					
Male	10	78	09	97	39
Female	10	121	21	152	61
Total	20	199	30	249	100
Year of study					
Year 1	07	52	06	65	26.1
Year 2	03	11	04	18	7.2
Year 3	03	38	03	44	17.7
Year 4	07	98	17	122	49.0
Total	20	199	30	249	100
Residential status					
Off-campus	04	110	13	127	51
Home with family	02	17	07	28	10.4
Off-campus (hostel)	05	24	04	33	13.3
On-campus	09	48	06	63	25.3
Total	20	199	30	249	100
Source of meals					
Restaurant	06	38	04	48	19.3
University cafeteria	05	21	02	28	11.2
Own preparation	09	140	24	173	69.5
Total	20	199	30	249	100

**Table 7:** Frequency of respondents' physical activity status.

Physical activity category				
Variable	Inactive (189)	Active (60)	*n* (249)	%
Age				
18–24	159	53	212	85.1
25–31	30	07	37	14.9
Sex				
Male	68	29	97	39
Female	121	31	152	61
Year of study				
Year 1	51	14	65	26.1
Year 2	15	03	18	7.2
Year 3	34	10	44	17.7
Year 4	89	33	122	49.0
Residential status				
Off-campus (rental)	94	33	127	51
Home with family	14	12	26	10.4
Off-campus (hostels)	28	05	33	13.3
On-campus	53	10	63	25.3
Walking minutes/day				
Less than 100	63	19	82	32.9
100 and above	126	41	167	67.1
Sedentary lifestyle/sitting hours				
< 8 h	93	32	125	50.2
≥ 8 h	96	28	124	49.8

## Data Availability

Data are available on request from the authors.
